# Membrane Fluidity and Temperature Sensing Are Coupled via Circuitry Comprised of Ole1, Rsp5, and Hsf1 in Candida albicans

**DOI:** 10.1128/EC.00138-14

**Published:** 2014-08

**Authors:** Michelle D. Leach, Leah E. Cowen

**Affiliations:** a Aberdeen Fungal Group, School of Medical Sciences, University of Aberdeen, Institute of Medical Sciences, Foresterhill, Aberdeen, United Kingdom; b Department of Molecular Genetics, University of Toronto, Toronto, Ontario, Canada

## Abstract

Temperature is a ubiquitous environmental variable which can profoundly influence the physiology of living cells as it changes over time and space. When yeast cells are exposed to a sublethal heat shock, normal metabolic functions become repressed and the heat shock transcription factor Hsf1 is activated, inducing heat shock proteins (HSPs). Candida albicans, the most prevalent human fungal pathogen, is an opportunistic pathogen that has evolved as a relatively harmless commensal of healthy individuals. Even though C. albicans occupies thermally buffered niches, it has retained the classic heat shock response, activating Hsf1 during slow thermal transitions such as the increases in temperature suffered by febrile patients. However, the mechanism of temperature sensing in fungal pathogens remains enigmatic. A few studies with Saccharomyces cerevisiae suggest that thermal stress is transduced into a cellular signal at the level of the membrane. In this study, we manipulated the fluidity of C. albicans membrane to dissect mechanisms of temperature sensing. We determined that in response to elevated temperature, levels of *OLE1*, encoding a fatty acid desaturase, decrease. Subsequently, loss of *OLE1* triggers expression of *FAS2*, encoding a fatty acid synthase. Furthermore, depletion of *OLE1* prevents full activation of Hsf1, thereby reducing *HSP* expression in response to heat shock. This reduction in Hsf1 activation is attributable to the E3 ubiquitin ligase Rsp5, which regulates *OLE1* expression. To our knowledge, this is the first study to define a molecular link between fatty acid synthesis and the heat shock response in the fungal kingdom.

## INTRODUCTION

Microorganisms inhabit dynamic environments in which they are continually exposed to environmental stimuli and stresses. Survival depends upon effective environmental response strategies that have been uniquely tuned over evolutionary time. By reacting to environmental changes via a sense and respond logic, cells continuously monitor their environment and coordinate appropriate cellular responses to specific stimuli (1). These cellular response strategies have been intensively studied for a variety of model organisms (2–5). Fundamentally, organisms utilize a myriad of signaling pathways that drive physiological adaptation to diverse environmental stresses, including temperature fluctuations and osmotic, oxidative, and weak acid stresses, as well as nutrient limitation (6, 7).

Temperature is a ubiquitous environmental variable, which can profoundly influence the physiology of living cells as it changes over time and space. When yeast cells are exposed to a sublethal heat shock, normal metabolic functions become repressed, and genes encoding heat shock proteins (HSPs) are induced (8). This induction occurs through the evolutionarily conserved heat shock transcription factor Hsf1. How the cell senses changes in ambient temperature, thus triggering Hsf1 activation, was thought to be driven by the accumulation of denatured proteins (9). However, a sustained heat shock would give rise to ongoing protein denaturation, leading to continual activation of HSPs. Instead, the transient nature of the heat shock response suggests that the thermal sensor becomes desensitized, allowing cells to adapt to a new basal level of activity (10, 11).

The cellular membrane serves as a direct sensor of its organism's environment. Its lipid structure is key to determining the physicochemical environment of the membrane, with the molecular packing of lipids acting as a direct determinant of membrane fluidity. At physiological temperatures, the fluidity of the membrane is increased through disorganized, unpacked unsaturated fatty acids, whereas saturated fatty acids remain tightly packed and retain a higher melting temperature (12, 13). Consequently, lipid saturation is regulated in response to changes in temperature. The fluidity of the membrane is governed by an intricate balance between saturated and unsaturated fatty acids. In the model yeast Saccharomyces cerevisiae, after the initial reaction of fatty acid synthesis, elongation of the carbon chain is catalyzed by the fatty acid synthases Fas1 and Fas2 to produce the saturated fatty acids palmitic acid (16:0) and stearic acid (18:0) (14). These are then converted to the monounsaturated fatty acids palmitoleic acid (16:1) and oleic acid (18:1) by the fatty acid Δ9 desaturase (Ole1), located at the surface of the endoplasmic reticulum (ER), by introducing double bonds at carbon 9 in the carbon chains (15).

The degree of unsaturation in the S. cerevisiae cellular membrane is highest at 15°C and lowest at 37°C, with fatty acid chains becoming longer with increasing temperature (16). But how does this affect the heat shock response? Studies on the fungal pathogen Histoplasma capsulatum established that addition of increasing concentrations of palmitic acid, a saturated fatty acid, paired with a temperature upshift upregulated transcription of the heat shock protein HSP82; conversely, addition of oleic acid, an unsaturated fatty acid, decreased heat shock gene transcription upon a rise in temperature (17). Furthermore, an S. cerevisiae mutant that lacks the fatty acid desaturase gene *OLE1* was complemented using constructs with the native (S. cerevisiae) *OLE1* promoter or the *OLE1* promoter from a temperature-tolerant or temperature-sensitive H. capsulatum strain, with the resulting strains each displaying differential expression of *OLE1* (18). Depending on the promoter used, complemented strains adjusted the physiology of the membrane by modifying the ratio of saturated to unsaturated fatty acids. Subsequently, each mutant displayed a different threshold temperature of heat shock gene expression. For instance, the temperature-sensitive strain complemented with the *OLE1* promoter that upregulates *OLE1* expression displayed a dramatic decrease in palmitic acid, as well as *HSP* expression (17).

Candida albicans, one of the most prevalent human fungal pathogens, is an opportunistic pathogen that has evolved as a relatively harmless commensal of the mucous membranes and digestive tracts of healthy individuals (19, 20). C. albicans frequently causes infections of mucosal membranes (thrush), and in immunocompromised patients this yeast can cause life-threatening systemic infections (19, 21). Even though C. albicans is obligately associated with warm-blooded mammals and occupies thermally buffered niches, it has retained the classic heat shock response (22). Indeed, Hsf1 is essential for viability in C. albicans, reflecting the fundamental importance of heat shock adaptation in all organisms (22–24). Our recent exploration of the dynamic regulation of Hsf1 during thermal adaptation has suggested that Hsf1 is activated even during slow thermal transitions such as the increases in temperature suffered by febrile patients (11). However, the mechanisms by which C. albicans senses changes in ambient temperature and activates Hsf1 remain an enigma.

In this study, we explore whether C. albicans utilizes membrane fluidity as a direct sensor of temperature. We determined that cells alter the levels of the fatty acid desaturase Ole1 in response to increased temperature and that loss of *OLE1* induces expression of *FAS2*, encoding a fatty acid synthase. We show that depletion of *OLE1* prevents full activation of Hsf1, contributing to a reduction in *HSP* expression in response to heat shock, and that this reduction in Hsf1 activation is attributable to the E3 ubiquitin ligase Rsp5, which regulates *OLE1* through the transcription factor Spt23. Therefore, we have established for the first time in the fungal kingdom a molecular link between membrane fluidity and the heat shock response.

## MATERIALS AND METHODS

### Strains and growth conditions.

All strains used are listed in Table 1. Strains were grown in YPD (1% yeast extract, 2% Bacto peptone, 2% glucose) (25). To impose an instant heat shock of 30°C to 42°C, cells were grown in YPD at 30°C to exponential phase and mixed with an equal volume of medium that had been prewarmed to 54°C in flasks that had been prewarmed at 42°C. Cells were grown at 42°C for the times indicated below. Doxycycline was added to YPD medium at a concentration of 1 μg/ml or 20 μg/ml.

**TABLE 1:** C. albicans strains used in this study

Strain	Genotype	Reference
SN95	*arg4*Δ*/arg4*Δ *his1*Δ*/his1*Δ *URA3/ura3*:: λ*imm434 IRO1/iro1*::λ*imm434*	50
CaLC2993	*arg4/arg4 his1/his1 URA3/ura3*::*imm434 IRO1/iro1*::*imm434 HSF1/HSF1-TAP-ARG4*	This study
CaLC3032	*arg4/arg4 URA3/ura3*::*imm434 IRO1/iro1*::*imm434 HIS/his1*::*CaTAR-FRT-NAT tetO-OLE1/ole1*Δ *HSF1/HSF1-TAP-ARG4*	This study
CaLC3366	*arg4/arg4 his1/his1 URA3/ura3*::*imm434 IRO1/iro1*::*imm434 rsp5*::*FRT CaTAR-FRT*::*tetO-RSP5 HSF1/HSF1-TAP-ARG4*	This study

### Strain construction.

To regulate oleic acid levels, one copy of the *OLE1* gene was deleted, and the other was placed under the control of the *tetO* promoter. Briefly, the NAT (nourseothricin) flipper cassette (pLC49) was amplified with oLC2798/oLC2799 containing regions of homology upstream and downstream of *OLE1*. The PCR product was transformed into the wild-type strain SN95 (CaLC239), and NAT-resistant transformants were PCR tested with oLC275/oLC2802 and oLC274/oLC2803 to verify integration of the cassette. The NAT cassette was then excised to create CaLC2851. The tetracycline-repressible transactivator, the *tetO* promoter, and the NAT flipper cassette were PCR amplified from pLC605 using oligonucleotides oLC2801 and oLC2798. The PCR product was transformed into CaLC2851. Correct upstream and downstream integration was verified by amplifying across both junctions by colony PCR using primer pairs oLC2802/oLC275 and oLC2804/oLC300, respectively. Loss of the wild-type band was verified using primer pair oLC2802/oLC2804. Hsf1 was then tagged in this strain as described below to create CaLC3032.

To regulate *RSP5*, one copy of the *RSP5* gene was deleted, and the other was placed under the control of the *tetO* promoter. Briefy, the NAT flipper cassette (pLC49) was amplified with oLC3359/oLC3360 containing regions of homology upstream and downstream of *RSP5*. The PCR product was transformed into the wild-type strain SN95 (CaLC239), and NAT-resistant transformants were PCR tested with oLC275/oLC3361 and oLC274/oLC3362 to verify integration of the cassette. The NAT cassette was then excised to create CaLC3032. The tetracycline-repressible transactivator, the *tetO* promoter, and the NAT flipper cassette were PCR amplified from pLC605 using oligonucleotides oLC3357/oLC3388. The PCR product was transformed into CaLC3302, and NAT-resistant transformants were PCR tested with oLC534/oLC3361 (upstream), oLC300/oLC3389 (downstream), and oLC3361/oLC3389 (wild type) to verify integration of the cassette, the NAT cassette was then excised. Hsf1 was then tagged in this strain as described below to create CaLC3366.

To determine Hsf1 phosphorylation status, Hsf1 was tagged with the TAP (tandem affinity purification) tag at its C terminus in the wild-type strain SN95 (creating CaLC2993) (Table 1) and in the *tetO-HSF1/hsf1*Δ strain to confirm functionality of the tagged allele, using a PCR-based strategy as described previously (26). Briefly, the tag and a selectable marker (*ARG4*) were PCR amplified from pLC573 (pFA-TAP-*ARG4* [27]) using oligonucleotides oLC2950/2922 (see Table S1 in the supplemental material). Fifty microliters of PCR product was transformed into C. albicans. Correct genomic integration was verified using appropriate primer pairs that anneal ∼500 bp upstream (oLC1597) or downstream (oLC1598) from both insertion junctions together with oLC1593 (TAP-R) and oLC1594 (*ARG4*-F), which target the TAP and the selectable marker (see Table S1).

### qRT-PCR.

To monitor gene expression changes in response to *OLE1* or *RSP5* depletion, strains SN95 (CaLC2993), CaLC3032 (*tetO-OLE1/ole1*Δ), and CaLC3366 (*tetO-RSP5/rsp5*Δ) were grown overnight at 30°C in YPD, with shaking at 200 rpm. Stationary-phase cultures were split and adjusted to an optical density at 600 nm (OD_600_) of 0.1; one culture was treated with doxycycline (BD Biosciences), while the other was left untreated. Cells were grown for 4 h at 30°C. To monitor gene expression changes in response to heat shock, wild-type (CaLC2993) and *tetO-OLE1/ole1*Δ (CaLC3032) cells were grown to mid-log phase and subjected to a 30°C to 42°C heat shock, and 50 ml was harvested from each culture at the specified time, centrifuged at 3,000 rpm for 2 min at 4°C, and washed once with distilled water (dH_2_O) before being frozen at −80°C. RNA was then isolated using the Qiagen RNeasy kit, and cDNA synthesis was performed using the AffinityScript cDNA synthesis kit (Stratagene). PCR was carried out using the SYBR green JumpStart *Taq* ReadyMix (Sigma-Aldrich) under the following cycle conditions: 95°C for 3 min, 95°C for 10 s, and 60°C for 30 s for 39 rounds, 95°C for 10 s, and 65°C for 5 s. All reactions were done in triplicate using the following primer pairs: for *HSP104*, oLC1620/oLC1621; for *HSP21*, oLC3217/oLC3218; for *OLE1*, oLC2805/oLC2806; for *RSP5*, oLC3408/oLC3409; and for *FAS2*, oLC3168/oLC3169. Transcript levels were normalized to *ACT1* (oLC2285/oLC2286) (see Table S1 in the supplemental material). Data were analyzed using Bio-Rad CFX Manager software, version 3.1 (Bio-Rad).

### Western blotting.

Total soluble protein was extracted and subjected to Western blotting using published protocols (28, 29). Briefly, mid-log-phase cells were pelleted by centrifugation, washed with sterile water, and resuspended in lysis buffer (0.1 M Tris-HCl [pH 8.0], 10% glycerol, 1 mM dithiothreitol [DTT], phenylmethylsulfonyl fluoride [PMSF], and protease inhibitor cocktail). An equal volume of 0.5-mm acid-washed beads was added to each tube. Cells were mechanically disrupted on a BioSpec (Bartlesville, OK) Mini-Beadbeater for six 30-s periods, with 1 min on ice between each cycle. The lysate was pelleted by high-speed centrifugation and the supernatant removed for analysis. Protein concentration was determined using a Bradford reagent (Sigma-Aldrich) assay. Protein samples were mixed with one-sixth volume of 6× sample buffer (0.35 M Tris-HCl, 10% [wt/wt] SDS, 36% glycerol, 5% β-mercaptoethanol, and 0.012% bromophenol blue). Between 2 μg and 30 μg of protein was loaded in wells of a 6% SDS-PAGE gel. Separated proteins were transferred to a polyvinylidene difluoride (PVDF) membrane for 1 h at 100 V at 4°C. Membranes were blocked in 5% milk in phosphate-buffered saline (PBS) containing 0.1% Tween 20 (PBS-T) at room temperature for 1 h and subsequently incubated in primary antibody as follows. All primary antibodies were left on the membrane for 1 h at room temperature. Membranes were washed with 1× PBS-T and probed for 1 h with secondary antibody dissolved in 1× PBS-T and 5% milk. Membranes were washed in PBS-T and signals detected using an ECL Western blotting kit as per the manufacturer's instructions (Pierce).

TAP-tagged Hsf1 was detected using a 1:5,000 dilution of anti-TAP tag rabbit polyclonal antibody (Thermo Scientific; CAB1001) in PBS-T plus 5% milk. To detect Act1, an anti-Act1 antibody was used (Santa Cruz Biotechnology; sc47778) at a 1:1,000 dilution in PBS-T plus 5% milk. To detect Hsp90, a 1:10,000 dilution of anti-Hsp90 antibody was used (courtesy of Bryan Larson) in PBS-T plus 5% milk.

## RESULTS

### *OLE1* is regulated by temperature.

**FIG 1:**
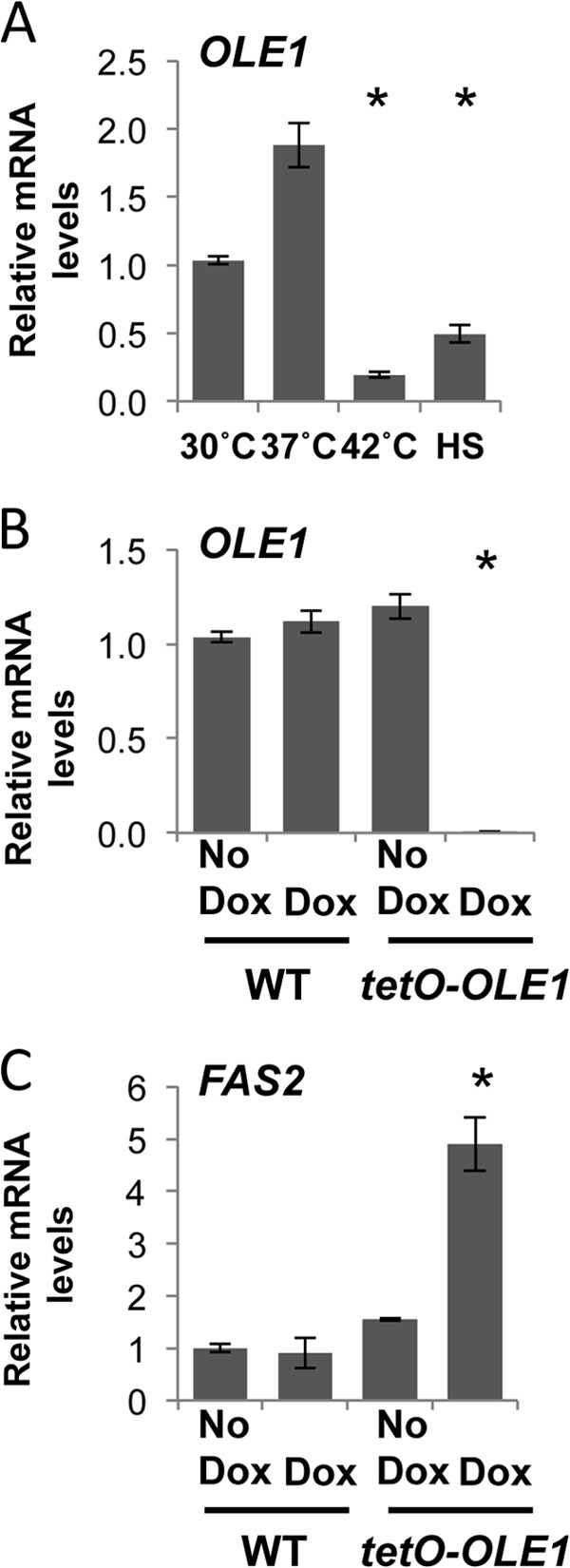
Membrane fluidity is tightly regulated in response to temperature and fatty acid ratios. (A) *OLE1* transcript levels decrease upon exposure to high temperatures. C. albicans wild-type (WT) cells were grown to exponential phase at 30°C, 37°C, or 42°C or subjected to a 15-min 30°C to 42°C heat shock (HS), and *OLE1* transcript levels were measured and normalized to the *ACT1* loading control. *, *P* < 0.05 compared to 30°C growth (Student *t* test). (B) *OLE1* transcript levels decrease following depletion in *tetO-OLE1/ole1*Δ cells. WT (CaLC2993 [Table 1]) and *tetO-OLE1/ole1*Δ cells (CaLC3032) were treated or not with 1 μg/ml of doxycycline (Dox) for 4 h, and *OLE1* transcript levels were measured by qRT-PCR and normalized to the *ACT1* loading control. *, *P* < 0.05 compared to the value for the wild type (Student *t* test). (C) Depleting Ole1 leads to an increase in *FAS2* transcript levels. WT and *tetO-OLE1/ole1*Δ cells were treated or not with 1 μg/ml of doxycycline for 4 h, and *FAS2* transcript levels were measured and normalized to the *ACT1* loading control. *, *P* < 0.05 compared to the value for the wild type (Student *t* test).

Mechanisms of temperature sensing in fungal pathogens remain an enigma, but evidence obtained with the benign yeast S. cerevisiae implicates membrane fluidity as a primary sensor of temperature (17). To establish whether temperature sensing and membrane fluidity are linked in the fungal pathogen C. albicans, which is obligately associated with warm-blooded mammals, we aimed to determine if *OLE1* expression is affected by temperature. As oleic acid is an unsaturated fatty acid, we hypothesized that during high-temperature growth, *OLE1* would be downregulated, promoting a less fluid membrane. Wild-type cells (CaLC2993) were grown at 30°C, 37°C, or 42°C for 4 h or subjected to a 30°C to 42°C heat shock before being harvested and snap-frozen. RNA was extracted, and expression of *OLE1* was determined relative to that of the housekeeping gene *ACT1* (Fig. 1A). As expected, levels of *OLE1* were significantly depleted when cells were heat shocked or grown at 42°C versus 30°C and 37°C (Fig. 1A). These data reinforce the notion that temperature affects fluidity of the membrane.

To elucidate the mechanisms of temperature sensing through membrane fluidity further, we created a conditional mutant with doxycycline-repressible expression of the fatty acid desaturase gene *OLE1*, which is known for its essentiality (30). One allele of *OLE1* was deleted, and the other was placed under the control of a tetracycline-repressible promoter. The addition of 1 μg/ml of the tetracycline analog doxycycline halted growth of the *tetO-OLE1/ole1*Δ strain after 6 h but had no effect on wild-type cells (data not shown). Expression of *OLE1* was measured by quantitative reverse transcription-PCR (qRT-PCR) after 4 h of growth in the absence or presence of 1 μg/ml of doxycycline, when cells were in mid-log phase (OD_600_ = 0.6) (Fig. 1B). The addition of doxycycline had no effect on *OLE1* expression in wild-type cells but caused a significant decrease in *OLE1* expression in the *tetO-OLE1/ole1*Δ strain (Fig. 1B).

Ole1 is responsible for introducing the double bond into saturated fatty acetyl coenzyme A (acetyl-CoA) substrates to produce monounsaturated fatty acids. After the initial reaction of fatty acid synthesis, which involves the incorporation of acetyl-CoA with CO_2_ to generate malonyl-CoA (31), elongation of the carbon chain is catalyzed by the fatty acid synthases Fas1 and Fas2 to produce long-chain saturated fatty acids such as palmitic and stearic acids (14); these act as the precursors for the subsequent desaturation reactions to produce monounsaturated fatty acids. We postulated that depletion of Ole1 would act as a signal for the production of saturated fatty acid precursors, essentially leading to an increase in the levels of the fatty acid synthases. To test this, we depleted *OLE1* as previously described and determined expression of the fatty acid synthase gene *FAS2* by qRT-PCR (Fig. 1C). As expected, loss of *OLE1* triggered a 5-fold increase in *FAS2* levels. Therefore, the cell responds to temperature by intricately regulating the levels of saturated and unsaturated fatty acids.

### Ole1 regulates components of the heat shock response.

**FIG 2:**
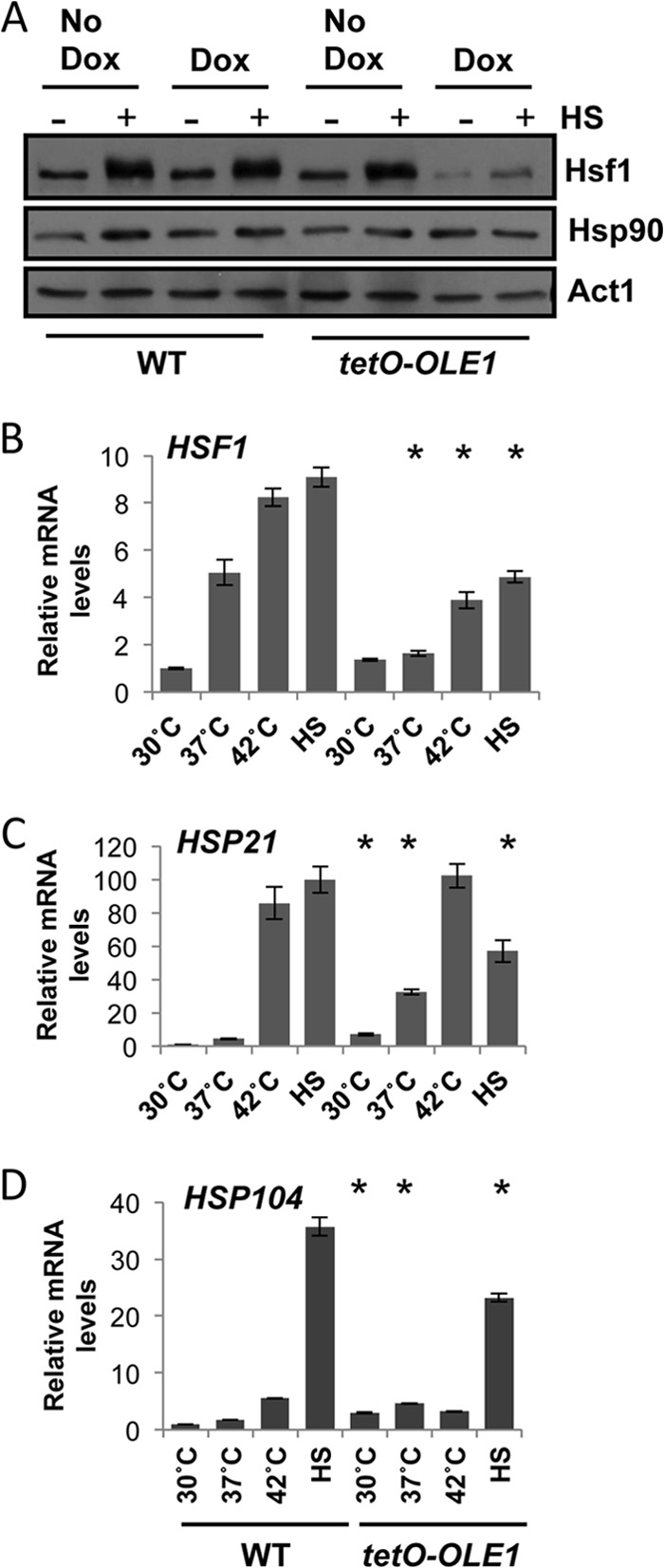
Depletion of Ole1 inhibits full activation of Hsf1, leading to a loss in *HSP* expression during high-temperature growth and heat shock. (A) Phosphorylation of C. albicans Hsf1 during a 30°C to 42°C heat shock, revealed by Western analysis. Exponentially growing WT (wild type, CaLC2993 [Table 1]) and *tetO-OLE1/ole1*Δ cells (CaLC3032) were treated or not with1 μg/ml of doxycycline for 4 h and subjected to a 15-min 30°C to 42°C heat shock. Protein was extracted immediately, and Hsf1 phosphorylation was monitored using an anti-TAP antibody recognizing *HSF1*-TAP. Membranes were stripped and reprobed for Hsp90, and actin served as an internal loading control. (B to D) Expression of *HSF1* (B), *HSP21* (C), and *HSP104* (D) during growth at 30°C, 37°C, or 42°C or after a 15-min 30°C to 42°C heat shock (HS), as determined by qRT-PCR of the corresponding transcripts relative to the internal *ACT1* mRNA control. *, *P* < 0.05 compared to the value for the wild type at the corresponding temperature (Student *t* test).

To determine whether a link exists between membrane fluidity and temperature sensing in C. albicans, we looked to components of the heat shock response. The heat shock transcription factor Hsf1 is rapidly activated by phosphorylation following a heat shock, leading to the upregulation of heat shock proteins (22). We hypothesized that if the membrane does indeed sense temperature, depletion of oleic acid would misregulate Hsf1 activation. Wild-type and *tetO-OLE1/ole1*Δ cells were grown in the absence or presence of 1 μg/ml of doxycycline for 4 h to deplete *OLE1*. Cells were either left untreated (30°C) or treated with a 15-min 30°C to 42°C heat shock. Protein was extracted, and Hsf1-TAP was visualized using an anti-TAP antibody (Fig. 2A). Wild-type cells in the absence and presence of doxycycline respond similarly, activating Hsf1 by phosphorylation, as seen by an upshift and broadening of the band corresponding to Hsf1 upon the thermal shock. The *tetO-OLE1/ole1*Δ strain also activates Hsf1 similarly to wild-type cells in the absence of doxycycline. However, in the presence of doxycycline, we observed a dramatic decrease not only in the phosphorylation status of Hsf1 but also in its protein levels. Based on this, we hypothesized that levels of the heat shock protein Hsp90, which is regulated by Hsf1, would be reduced. However, when we probed the blot with an anti-Hsp90 antibody, Hsp90 levels remained constant. This is most likely due to the stability of Hsp90 (M. D. Leach, unpublished data).

Next, we aimed to determine if the impact of *OLE1* depletion on Hsf1 levels manifests exclusively at the translational level, or if it is also evident at the transcriptional level. Expression of *HSF1* was measured in wild-type cells and the *tetO-OLE1/ole1*Δ cells in the presence of 1 μg/ml of doxycycline after growth at 30°C, 37°C, or 42°C or upon a 15-min 30°C to 42°C heat shock (Fig. 2B). In wild-type cells, *HSF1* levels increased up to 8-fold with increasing temperature and rapidly increased upon heat shock, equivalent to levels of cells grown at 42°C. However, loss of *OLE1* hampers *HSF1* expression during high-temperature growth, with levels increasing only 3-fold at 42°C. Additionally, when cells are exposed to a heat shock, *HSF1* expression is only half that of wild-type cells. Doxycycline has no effect on *HSF1* expression (see Fig. S1 in the supplemental material). These data provided the first molecular link between membrane fluidity and temperature sensing in fungi.

Our next goal was to ascertain if misregulation of Hsf1 upon *OLE1* depletion affects expression of key heat shock proteins involved in the heat shock response. Under the same conditions as used to determine *HSF1* levels, we monitored expression of *HSP104* and *HSP21*, which encode two heat shock proteins essential for thermal adaptation in C. albicans (22, 32). We made three observations. First, there was an increase in both *HSP104* and *HSP21* under basal conditions upon *OLE1* depletion (Fig. 2C and D). This increase remained at 37°C, with both genes presenting significantly higher expression in the *tetO-OLE1/ole1*Δ strain than in its wild-type counterpart. Therefore, depletion of *OLE1* promotes the induction of *HSP* expression in the absence of any heat stress. Second, depletion of *OLE1* had little impact on expression of the *HSP*s at 42°C. This could be due to a balance between the initial upregulation of these *HSP* genes in response to exposure to 42°C, before full depletion of Ole1, and stability of the *HSP* genes during high-temperature growth. Third, upon treatment with a 30°C to 42°C heat shock, *HSP* expression increased 100-fold for *HSP21* (Fig. 2C) and 35-fold for *HSP104* (Fig. 2D) in wild-type cells but reached only half of wild-type levels when *OLE1* was depleted. Again, addition of doxycycline to wild-type cells had no effect on *HSP* expression (Fig. S1). In summary, depletion of *OLE1* reduces Hsf1 protein levels and Hsf1 activation upon heat shock. In addition, *HSF1* and *HSP* levels are misregulated at the transcriptional level.

### Rsp5 regulates expression of *OLE1* and activation of Hsf1.

The promoter of *S. cerevisiae OLE1* contains multiple transcriptional regulatory elements, allowing for the elaborate control of the levels of unsaturated fatty acids. Transcription can be repressed through the addition of unsaturated fatty acids and activated in response to low temperature and hypoxia via the ER-bound transcription factors ScSpt23p and ScMga2p (33). Both factors are, in turn, activated by ubiquitin/proteasome-dependent ER-associated degradation, through the E3 ubiqutin ligase ScRsp5 (34). C. albicans retains only one homolog of the S. cerevisiae functionally redundant gene, *CaSPT23*. Repression of *SPT23* in C. albicans blocks expression of *OLE1* (35), suggesting that Rsp5 could be the upstream element that transduces signals pertaining to temperature changes sensed at the membrane.

**FIG 3:**
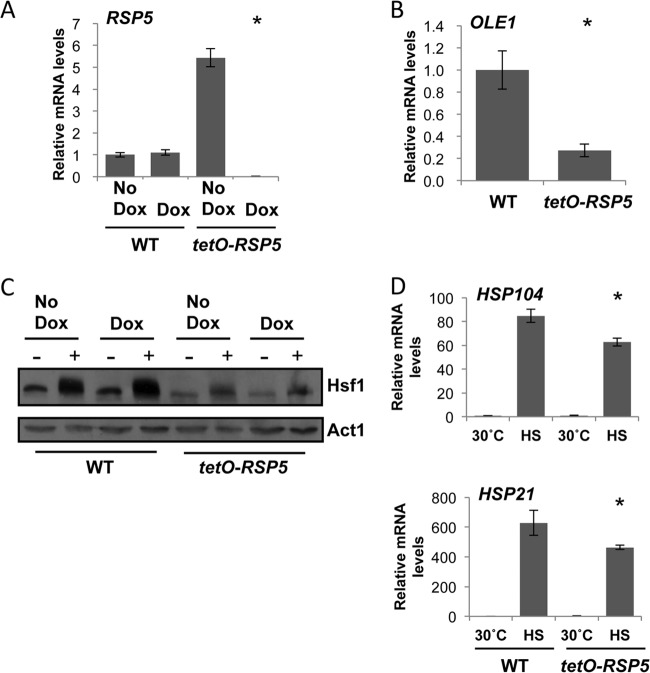
Rsp5 regulates Hsf1 in part through *OLE1*. (A) *RSP5* transcript levels are upregulated in *tetO-RSP5/rsp5*Δ strain in the absence of doxycycline and depleted in the presence of doxycycline. WT (wild type, CaLC2993 [Table 1]) and *tetO-RSP5/rsp5*Δ (CaLC3366) cells were treated or not with 20 μg/ml of doxycycline overnight for 18 h and then subcultured the following morning and grown for a further 5 h under the same conditions. *RSP5* transcript levels were normalized to the *ACT1* loading control. (B) Depleting Rsp5 causes a decrease in *OLE1* transcript levels. WT and *tetO-RSP5/rsp5*Δ cells were treated or not with 20 μg/ml of doxycycline as stated above and *OLE1* transcript levels were measured and normalized to the *ACT1* loading control. (C) Rsp5 is required for Hsf1 expression and full activation. WT and *tetO-RSP5/rsp5*Δ cells were treated or not with 20 μg/ml of doxycycline as stated above, exposed to a 15-min 30°C to 42°C heat shock, and subjected to Western analysis. Decreased Hsf1-TAP levels were observed in the *tetO-RSP5/rsp5*Δ strain. Membranes were stripped and reprobed for actin, which served as the internal loading control. (D) Expression of *HSP21* and *HSP104* during growth at 30°C or after a 15-min 30°C to 42°C heat shock (HS), as determined by qRT-PCR relative to the internal *ACT1* mRNA control. *, *P* < 0.05 in *tetO-RSP5/rsp5*Δ cells compared to wild-type cells (Student *t* test).

We hypothesized that Rsp5 regulates *OLE1* expression and hence Hsf1 activation in C. albicans. To test this, we constructed a tetracycline-repressible *RSP5* conditional expression strain, in which *RSP5* expression is repressed upon the addition of doxycycline. Addition of 20 μg/ml of doxycycline for prolonged periods did not affect the viability of the *tetO-RSP5/rsp5*Δ strain, suggesting that *RSP5* is not an essential gene (data not shown). To ensure full depletion of *RSP5*, *tetO-RSP5/rsp5*Δ cells were subcultured into YPD in the presence or absence of 20 μg/ml of doxycycline for 18 h and then subcultured again the following morning under the same conditions and grown to mid-log phase (approximately 5 h). RNA was extracted for qRT-PCR analysis of *RSP5* transcript levels (Fig. 3A). As expected, *RSP5* was significantly decreased upon addition of 20 μg/ml of doxycycline. We also noted that in the absence of doxycycline, *RSP5* levels in the *tetO-RSP5/rsp5*Δ strain were significantly higher than in the wild-type counterpart, suggesting that the *tetO* promoter is stronger than the native promoter. To determine if Rsp5 regulates *OLE1* expression, we depleted *RSP5* at 30°C overnight and until mid-log phase and examined *OLE1* expression by qRT-PCR. Notably, *OLE1* levels were 4-fold lower upon *RSP5* depletion than in wild-type cells (Fig. 3B), indicating that Rsp5 regulates *OLE1*, likely through the transcription factor Spt23 (35). Even though *RSP5* expression was significantly increased in the absence of doxycycline (Fig. 3A), *OLE1* expression remained the same as in wild-type cells (see Fig. S2A in the supplemental material).

Given that we can manipulate *RSP5* expression and thereby reduce levels of *OLE1*, we wanted to examine the effect of *RSP5* depletion on Hsf1 activity in response to heat shock. Wild-type cells and *tetO-RSP5/rsp5*Δ cells were grown in the absence or presence of 20 μg/ml of doxycycline as previously described and either kept at 30°C or exposed to a 15-min 30°C to 42°C heat shock. Proteins were extracted and subjected to Western analysis to visualize Hsf1 activation (Fig. 3C). As predicted, doxycycline-mediated depletion of *RSP5* led to reduced levels of Hsf1 activation, as indicated by a decrease in phosphorylation with a narrower band and loss of a band shift. We also noted that in the absence of doxycycline, although a visible shift is seen in the presence of a heat shock in the *tetO-RSP5/rsp5*Δ strain, the levels of Hsf1 are significantly lower than in the wild-type counterpart, suggesting that the misregulation of *RSP5* affects Hsf1 protein levels.

In order to confirm that Hsf1 activation upon depletion of *RSP5* is not equivalent to that seen in wild-type cells upon a heat shock, we monitored expression of *HSP104* and *HSP21* under the same conditions (Fig. 3D). As expected, expression of both *HSP*s increased significantly upon heat shock; however, this increase was reduced when *RSP5* was depleted. Expression of *HSP* genes was not affected by doxycycline in the wild-type strain (see Fig. S2B and C in the supplemental material). Although expression of *HSP104* and *HSP21* was significantly lower upon depletion of *RSP5*, it was not reduced to the same extent as that observed upon depletion of *OLE1* (Fig. 3C and D). In part, this may be because depletion of *RSP5* does not fully deplete *OLE1* (Fig. 3A), even in the presence of heat shock (Fig. S2D). Therefore, depletion of *RSP5* phenocopies loss of *OLE1*.

## DISCUSSION

In this paper, we have addressed one of the most fundamental questions in biology: how do cells sense temperature? The ability to sense the surrounding temperature is key for virulence. Indeed, many fungal pathogens inhabit a remarkable diversity of environments; for example, Cryptococcus neoformans, Histoplasma capsulatum, and Aspergillus fumigatus are found in diverse environments such as pigeon excreta and soil, but each retains the ability to grow at 37°C. The loss of genes necessary for high-temperature growth in these pathogens results in attenuated virulence and at times even death (36–38), suggesting the importance of temperature sensing for pathogenesis. Besides governing virulence, temperature regulates numerous cellular processes that often contribute to pathogenesis. For example, morphological transitions in C. albicans are controlled by temperature. At ambient temperature, this organism favors the yeast form, while elevated temperatures induce filamentous growth, enabling penetration of the epithelium (39). C. albicans can also switch from a white to opaque cellular state in host niches with lower temperatures, such as skin, facilitating mating (40).

By manipulating C. albicans membrane composition, we have begun to understand the ways in which fungal pathogens sense temperature. Using a tetracycline-repressible promoter, we were able to deplete levels of the fatty acid desaturase gene *OLE1*, which regulates synthesis of the monounsaturated fatty acid oleic acid. In doing so, we triggered a 5-fold increase in the fatty acid synthase gene *FAS2* (Fig. 1C). These data suggest that the cell compensates for the loss of unsaturated fatty acids by increasing the levels of *FAS2* in an attempt to increase levels of saturated fatty acids, which are the precursors for unsaturated fatty acids. Furthermore, heat shock or high-temperature growth caused a significant decrease in *OLE1* expression (Fig. 1A). This is consistent with previous studies with S. cerevisiae illustrating a 4- to 6-fold increase in *OLE1* mRNA when cells were rapidly shifted from 30°C to 10°C (41). These data reinforce the notions that temperature affects fluidity of the membrane and that the cell continually monitors the ratio of saturated to unsaturated fatty acids.

Previous studies with the fungal pathogen H. capsulatum and the model yeast S. cerevisiae have suggested a role for the membrane in sensing the surrounding temperature. Indeed, Carratù and colleagues observed a strong increase in *HSP* expression in H. capsulatum when cells were heat shocked at 37°C upon the addition of external saturated fatty acids into the media. Addition of unsaturated fatty acids, however, reduced *HSP* expression during the same heat shock (17). Furthermore, Chatterjee and colleagues demonstrated a 9°C increase in the optimal activation of the heat shock response upon supplementation of S. cerevisiae with unsaturated fatty acids (42). Therefore, it would appear that the ratio of saturated to unsaturated fatty acids of the membrane is a key determinant in the perception of rapid temperature changes. Consistent with these data, we discovered that loss of *OLE1* caused upregulated expression of key components of the heat shock response during growth at 30°C and 37°C, but this upregulation was lost upon high-temperature growth or exposure to a short heat shock (Fig. 2B to D). Indeed, based on our findings and those of others, it would appear that upon loss of *OLE1*, levels of unsaturated fatty acids are severely depleted within the membrane, prompting the cell to assume that the temperature has increased. In turn, *HSP* expression is increased at 30°C and 37°C. However, if the ratio of fatty acids is acting as the primary sensor of temperature, upon subjecting the cell to a drastic heat shock, the temperature change in a membrane that is depleted of unsaturated fatty acids is not properly recognized, thereby preventing full activation of Hsf1 and reducing upregulation of *HSP* genes (Fig. 2).

One of the conundrums surrounding temperature sensing is the remarkable speed at which cells are able to sense and respond to the surrounding environment. C. albicans is able to activate Hsf1 within 1 min of exposure to a heat shock (11), suggesting that the sensor itself may reside in the plasma membrane, becoming activated upon slight changes in membrane fluidity. This would then be transduced into a signal that could further regulate fatty acid expression. Based on this notion, we examined regulators of fatty acid synthesis. In S. cerevisiae, *OLE1* expression is regulated via the ER-bound transcription factors Spt23 and Mga2 (33), which are activated by the E3 ubiquitin ligase Rsp5 (34). C. albicans has retained only one homolog of the S. cerevisiae functionally redundant genes, *CaSPT23*. Oh and colleagues found that upon repression of *SPT23* in C. albicans, expression of *OLE1* was blocked (35). Therefore, we postulated that Rsp5 regulates expression of *OLE1*, through Spt23, thereby acting as a downstream element that is activated upon changes that occur in the plasma membrane and subsequently regulating activation of the heat shock response. By regulating the levels of Rsp5 using the tetracycline-repressible promoter (Fig. 3A), we noted a significant decrease in *OLE1* expression upon loss of *RSP5* (Fig. 3B). This translated to a reduction in Hsf1 activation during a short heat shock (Fig. 3C), similar to that seen upon loss of *OLE1* (Fig. 2A). Furthermore, this decrease in Hsf1 activation contributed to a decrease in *HSP21* and *HSP104* expression upon a heat shock (Fig. 3D).

We also observed that in the absence of doxycycline in the *tetO-RSP5/rsp5*Δ strain, *RSP5* levels are significantly higher than in wild-type cells (Fig. 3A; see also Fig. S2E in the supplemental material). When we looked at Hsf1 activation under these conditions, although Hsf1 is activated and *HSP* expression remains similar to that observed in wild-type cells (see Fig. S2B and C), Hsf1 levels are much lower than in the wild-type counterpart and similar to those observed upon Rsp5 depletion. For S. cerevisiae, two recent studies by Haitani and colleagues show that an Rsp5 mutant, *rsp5*^A410E^, exhibits a decrease in Hsf1 levels compared to that for wild-type cells but relatively similar *HSF1* mRNA levels. Further investigation showed accumulation of *HSF1* mRNA in the nucleus, suggesting that Rsp5 is required for *HSF1* nuclear export (43, 44). Our data suggest that a similar mechanism operates in C. albicans, whereby misregulation of Rsp5 prevents proper export of *HSF1* mRNA. However, depletion of *OLE1* does reduce *HSF1* expression, suggesting more complex circuitry connecting Rsp5-Ole1-Hsf1.

These data provide evidence for the first inhibitor of Hsf1 activation in response to a heat shock. The loss of the unsaturated fatty acid desaturase gene *OLE1* drastically increases the fatty acid synthase gene *FAS2* (Fig. 1C), leads to an upregulation of *HSP* genes in the absence of heat shock but inhibits full *HSP* expression upon heat shock (Fig. 2C and D). This inhibition is likely due to a reduction in Hsf1 activation (Fig. 2A). This suggests that membrane fluidity is a key sensor of temperature; however, before changes can occur in levels of fatty acids, a signal must be transduced. *OLE1* is regulated by the transcription factor Spt23, which is regulated by the E3 ubiquitin ligase Rsp5. We discovered that loss of *RSP5* phenocopies loss of *OLE1*, suggesting that Rsp5 could be one of the early sensors of temperature, which coordinately regulates fatty acid synthesis and activation of the heat shock response.

Understanding circuitry governing temperature sensing offers therapeutic opportunities for crippling diverse microbial pathogens. In the context of Candida species, many are human commensals and able to disseminate into the bloodstream, causing systemic infections. Inhibiting fatty acid biosynthesis would be an ideal way to eradicate Candida species from the mycobiome prior to immunosuppressive treatments that render patients vulnerable to infection. Importantly, fungal Ole1 is comprised of two domains: the N and C domains, containing the catalytic motif for the desaturase activity and the cytochrome *b*_5_ activity, respectively. In contrast, humans have two separate enzymes to carry out these activities, making fungal Ole1 an ideal antifungal target (33). Furthermore, recent studies illustrated that components of fatty acid biosynthesis from different Candida species are essential for establishing and maintaining infection in a mouse model of candidiasis (45–47). Xu and colleagues demonstrated that C. albicans Ole1 is necessary for virulence, and Nguyen and colleagues obtained similar results with C. parapsilosis. This is consistent with our findings that loss of Ole1 leads to a significant decrease in Hsf1 activation (Fig. 2A) and a previous study showing that activation of Hsf1 is necessary for virulence in C. albicans (48). Finally, Krishnamurthy and colleagues discovered that depletion of *OLE1* blocks one of the key virulence traits in C. albicans, hyphal development, when cells are grown under aerobic conditions (30). This study provides us with the first mechanistic link between fatty acid synthesis and the heat shock response in the fungal kingdom, highlighting Ole1 as an attractive antifungal. Beyond fungal pathogens, fatty acid biosynthesis is emerging as a powerful target for the development of antibacterial agents, with small-molecule inhibitors currently in clinical use to treat tuberculosis and as ubiquitous consumer antimicrobials (49).

## Supplementary Material

Supplemental material
