# Supplementary material for: Membrane Fluidity and Temperature Sensing Are Coupled via Circuitry Comprised of Ole1, Rsp5, and Hsf1 in Candida albicans
Source: Eukaryot Cell. 2014 Aug;13(8):1077–84. doi: 10.1128/EC.00138-14 (PMC4135801; doi:10.1128/EC.00138-14)
Supplement: Supplemental material [file supp_13_8_1077__index.html]

Membrane Fluidity and Temperature Sensing Are Coupled via Circuitry Comprised of Ole1, Rsp5, and Hsf1 in Candida albicans — Supplemental material 

# Membrane Fluidity and Temperature Sensing Are Coupled via Circuitry Comprised of Ole1, Rsp5, and Hsf1 in Candida albicans

## Supplemental material

**Files in this Data Supplement:**

- Supplemental file 1 -

  Fig. S1 and S2 and Table S1.

  PDF, 293K
