# Supplementary material for: Membrane Fluidity and Temperature Sensing Are Coupled via Circuitry Comprised of Ole1, Rsp5, and Hsf1 in Candida albicans
Source: Eukaryot Cell. 2014 Aug;13(8):1077–84. doi: 10.1128/EC.00138-14 (PMC4135801; doi:10.1128/EC.00138-14)
Supplement: Supplemental material [file EC.00138-14_zek999094317so1.pdf]

Supplemental Figure Legends

Figure S1: *HSF1*, *HSP21* and *HSP104* expression upon depletion of *OLE1* in *C. albicans tetO-OLE1*/*ole1*Δ cells at different growth temperatures. WT (wild type, CaLC2993: Table 1) and doxycycline-conditional *C. albicans tetO-OLE1/ole1*Δ cells (CaLC3032) were treated with 0 or 1 μg/ml doxycycline, and expression of *HSF1*, *HSP21* and *HSP104* was monitored by qRT-PCR during growth at 30˚C, 37˚C, 42˚C or after a 15 minute 30˚C to 42˚C heat shock (HS), relative to the internal *ACT1* mRNA control.

Figure S2: Rsp5 depletion affects *OLE1* and *HSP* expression. (A) qRT-PCR of WT (wild type, CaLC2993: Table 1) and doxycycline-conditional *C. albicans tetO-RSP5/rsp5*Δ cells (CaLC3366) treated with 0 or 20 μg/ml doxycycline, illustrate a loss of *OLE1* transcript upon depletion of *RSP5*. Data relative to the *ACT1* mRNA control. *HSP104* (B), *HSP21* (C), *OLE1* (D) and *RSP5* (E) transcript levels in WT and *tetO-RSP5/rsp5*Δ cells treated with 0 (-) or 20 μg/ml (+) doxycycline for 18 + 5 hours at 30˚C or after a 15 minute 30˚C to 42˚C heat shock (HS), relative to the internal *ACT1* mRNA control.

Supplementary data Figure 1: Leach *et al.*

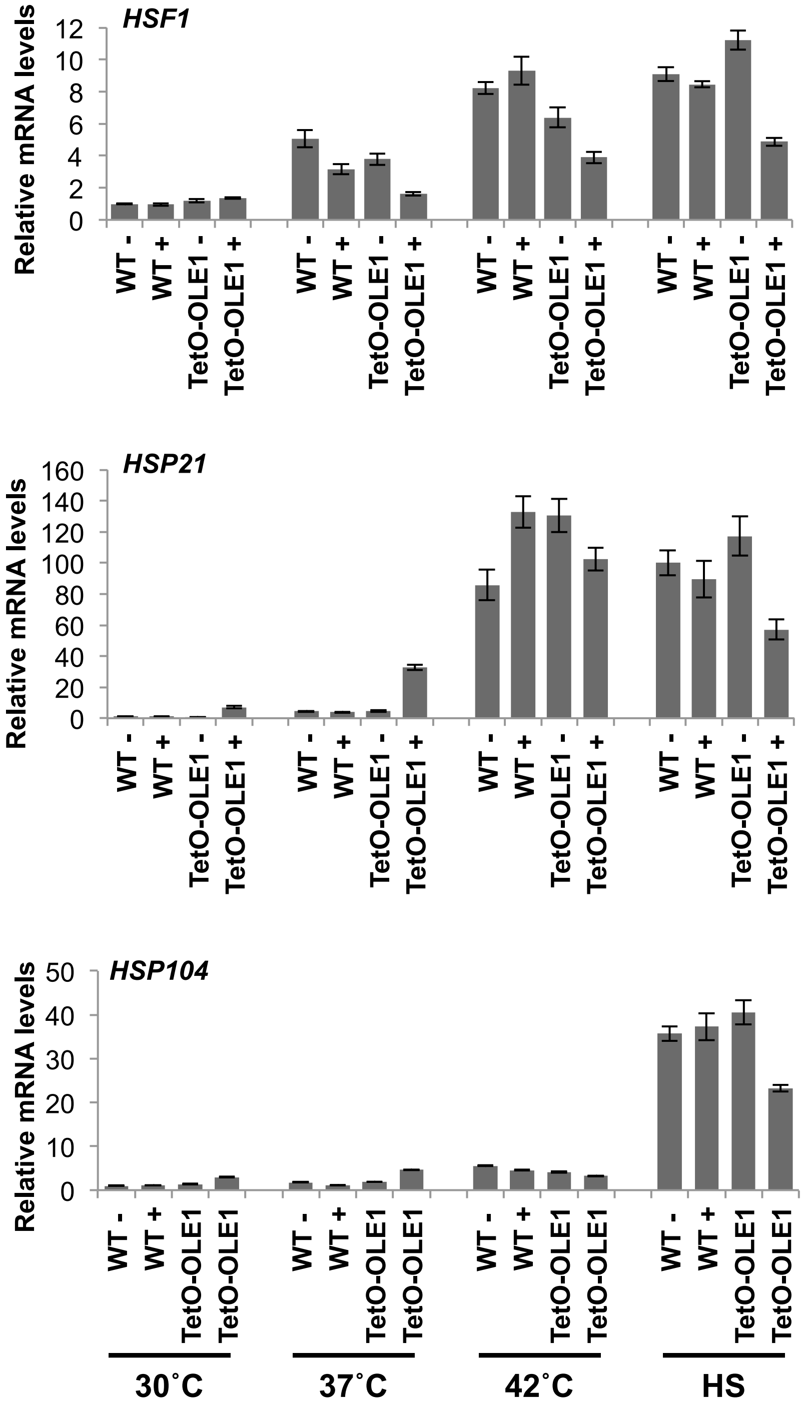

Supplementary data Figure 2: Leach *et al.*

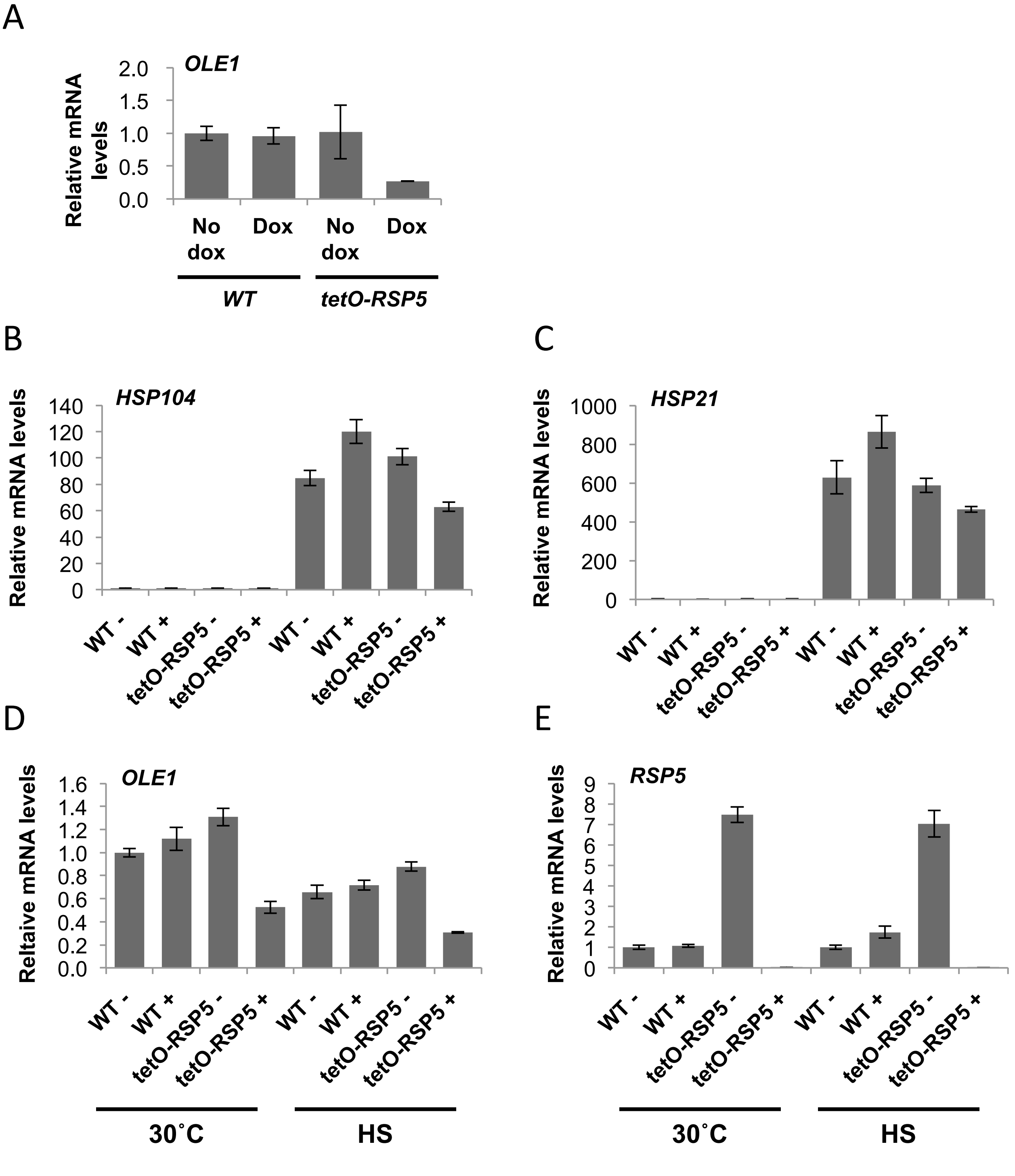

Table S1: Primers used in this study.

oLC2798	CaOLE1-NAT1 F1	TCGATCAAGTAACACATACTGTTATTACAAGAACTTGAAAGTCAAGACCAAAACAAATTAGCATTTCACAGGAAACAGCTATGACCATG
oLC2799	CaOLE1-NAT1 R1	AAACAAAAATGACAGAAAATGAAAACGAAAAATGAAAGAAAACAAATCAACCCAGCAATTTTCTATTTGCGTAAAACGACGGCCAG
oLC2802	CaOle1d Ext F (-306 bp)	GTATCAGAGGCAAAGTAATG
oLC2803	CaOle1d Ext R (+1667 bp)	CTTACAGACACTGAACTTG
oLC274	pJK863down-F	CTGTCAAGGAGGGTATTCTGG
oLC275	pJK863up-R	AAAGTCAAAGTTCCAAGGGG
oLC2801	CaOLE1-NAT1 pLC330 R1	TATTAGTACCAGCAGCAATGGCATTCAATTTAGTGATATCAACAGTTTCAAGTTGTTCAACTGTAGTCATcgactatttatatttgtatg
oLC2804	CaOle1d Int R (+125 bp)	CAGTGACCTTAACTAAATCG
oLC300	Tetp-F-NotI	ATAAGAATGCGGCCGCGTTTGGTTCAGCACCTTGTCG
oLC3359	CaRSP5-NAT1 F2	TTCTCAGTTGAGTTGAGTTGAGGTTCAACATTCATTTGTCACTCACTCACTCACTCCCCTTCTTCCCTTTGGAAACAGCTATGACCATG
oLC3360	CaRSP5-NAT1 R2	CAAACAAATATAAGTATATGTATGTATGTATATATAAATATAAATGTATAACCAGAAAGAAAGAAAGGTAAAACGACGGCCAG
oLC3361	CaRSP5d Ext F (-320 bp)	GCTGTTTCGGTTGTAATTAG
oLC3362	CaRSP5d Ext R (+2775 bp)	CAACGTCAACTCTTCAAATC
oLC3388	CaRSP5_TetO_pLC605	CAGCAACAACTTTAACGTTGATGGTGGTAGTATTGTTGTTGCTCCTTGATGTAGATATATTAGGAGCCATcgactatttatatttgtatg
oLC3389	CaRSP5+542 R	GTTGAGTTTGCAGTAGTAG
oLC3357	CaRSP5-NAT1 F1	TATACCATTCAATTTCTTCCATTTGTTCAACCTTCCCATCAATCGAAGAAAACTCTGTGTTGTATAAATTGGAAACAGCTATGACCATG
oLC534	CaTAR-797-R	gatggagatagtttacgg
oLC2922	CaHsf1-TAP ARG R	TATACCTAAACAACGAAAATAATGAATTGAAAAATATAATGATTATAGACACACTTCATCTATATCCTATtcgatgaattcgagctcgtt
oLC2950	CaHsf1-TAP-ARG4-F	TAACATAAATGATTCTAATGGTAATGAGAAGAAACTGAAGAAAAGATCAATCGAAGAAG

		TTAGCGATCATggtcgacggatccccgggtt
oLC1593	TAP-R	TAAACTTTGGATGAAGGCG
oLC1594	ARG4-F	ATGTTGGCTACTGATTTAGCTG
oLC1597	CaHsf1+1978-F	GGACAATCAATTCAGCAAGTG
oLC1598	CaHsf1+2607-R	GCATTTACTCTCAATAGAGTG
oLC1620	Hsp104+1402-F	CGAGCTAGTCATGAACAATTG
oLC1621	Hsp104+1660-R	GGATACCAGTCAATCTAGCA
oLC3217	CaHSP21+90-F	CCAAGAAAAATTGCTCAAGG
oLC3218	CaHSP21+325-R	CTTCATCAGGACTCAAATTC
oLC2805	CaOle1 RT-PCR F (+353 bp)	GACTCTAGTGTTGACATTTG
oLC2806	CaOle1 RT-PCR R (+537 bp)	GTGAATACGATGAGAATGAC
oLC3408	CaRSP5+1077 F	GGTTGGGAACAAAGATTTAC
oLC3409	CaRSP5+1273 R	GAGCAGTATTGGTTAATCTC
oLC3168	CaFAS2+438-F	CAAGGCTAACTTGTTAATCC
oLC3169	CaFAS2+658-R	GAAGTCTTTCCTAATTGACC
oLC2285	CaACT1+855-F	GACCTTGAGATACCCAATTG
oLC2286	CaACT1+1076-R	CAGCTTGAATGGAAACGTAG
